# HIV Infection Drives Foam Cell Formation via NLRP3 Inflammasome Activation

**DOI:** 10.3390/ijms25042367

**Published:** 2024-02-17

**Authors:** Maurizio Caocci, Meng Niu, Howard S. Fox, Tricia H. Burdo

**Affiliations:** 1 Department of Microbiology, Immunology, and Inflammation, Center for Neurovirology and Gene Editing, 3500 N Broad St. MERB 760, Lewis Katz School of Medicine at Temple University, Philadelphia, PA 19140, USA; tuh20265@temple.edu; 2 Department of Neurological Sciences, University of Nebraska Medical Center, Omaha, NE 68198, USA; meng.niu@unmc.edu (M.N.); hfox@unmc.edu (H.S.F.)

**Keywords:** HIV, oxLDL, foam cells, NLRP3 inflammasome, cardiovascular disease, PWH

## Abstract

Persistent immune activation is linked to an increased risk of cardiovascular disease (CVD) in people with HIV (PWH) on antiretroviral therapy (ART). The NLRP3 inflammasome may contribute to elevated CVD risk in PWH. This study utilized peripheral blood mononuclear cells (PBMCs) from 25 PWH and 25 HIV-negative controls, as well as HIV in vitro infections. Transcriptional changes were analyzed using RNAseq and pathway analysis. Our results showed that in vitro HIV infection of macrophages and PBMCs from PWH had increased foam cell formation and expression of the NLRP3 inflammasome components and downstream cytokines (caspase-1, IL-1β, and IL-18), which was reduced with inhibition of NLRP3 activity using MCC950. Transcriptomic analysis revealed an increased expression of multiple genes involved in lipid metabolism, cholesterol storage, coronary microcirculation disorders, ischemic events, and monocyte/macrophage differentiation and function with HIV infection and oxLDL treatment. HIV infection and NLRP3 activation increased foam cell formation and expression of proinflammatory cytokines, providing insights into the mechanisms underlying HIV-associated atherogenesis. This study suggests that HIV itself may contribute to increased CVD risk in PWH. Understanding the involvement of the inflammasome pathway in HIV atherosclerosis can help identify potential therapeutic targets to mitigate cardiovascular risks in PWH.

## 1. Introduction

Persistent immune activation likely plays a role in the increased risk of cardiovascular disease (CVD) observed in people with human immunodeficiency virus (HIV) (PWH) who are on antiretroviral therapy (ART) [1]. PWH who are untreated show endothelial dysfunction that improves with ART but does not return to normal levels in the short term [2,3,4]. One possible factor contributing to this elevated inflammation is the inflammasome, an innate immune complex that controls the immunological environment by regulating proinflammatory cytokines. The inflammasome could potentially serve as a mediator of cardiovascular disease (CVD) risk in PWH, as it has been implicated in both HIV pathogenesis and CVD in the general population. Nevertheless, there are limited data concerning the correlation between inflammasome activation and coronary plaque indices in PWH [5]. 

HIV infection itself acts as a trigger for inflammasome activation [6]. The NLRP3 inflammasome, a multiprotein complex that recruits pro-caspase-1 via ASC (adapter protein apoptosis-associated speck-like protein containing a CARD), crucial for the processing and release of IL-1β and IL-18 and amplifying the inflammatory cascade. The significance of IL-1B and IL-18 in the context of NLRP3 inflammasome activation is underscored by their involvement in various inflammatory conditions, including infectious diseases and autoimmune disorders [7,8,9]. The involvement of caspase-1 in pyroptosis, an inflammatory form of cell death, is a significant factor contributing to CD4+ T-cell depletion. This depletion, along with immune exhaustion, might partially explain the association of HIV progression with a low CD4/CD8 ratio, an indication of poor immune health [10]. 

Microbial Pathogen-Associated Molecular Patterns (PAMPs) play a crucial role in the pathogenesis of HIV-associated inflammation. Bacterial lipopolysaccharides (LPS) are among the notable PAMPs that can exacerbate inflammation in PWH. The presence of LPS, derived from bacterial cell walls, has been associated with immune activation and progression of HIV disease. Furthermore, co-infections, such as cytomegalovirus (CMV) and Epstein–Barr virus (EBV), introduces additional PAMPs that can influence the inflammatory milieu in PWH. These herpesviruses are known to elicit immune responses and contribute to chronic inflammation, potentially impacting the progression of HIV disease [11,12].

The danger signals initiated by HIV consist of aggregated host molecules, including crystalline cholesterol or calcium precipitates, tissue damage- and cell death-associated molecules, such as extracellular matrix components, heat shock proteins, alarmins, and adenosine triphosphate (ATP), as well as modified host molecules, including advanced glycation end products and oxLDL (oxidized LDL). In particular, the finding that oxLDL and crystalline cholesterol, which are abundantly present in atherosclerotic lesions, represent potent inflammatory molecules, provided a link between vascular cholesterol deposition and vascular inflammation. Foam cells, critical players in atherosclerosis, develop through the accumulation of lipid-laden macrophages within the arterial walls. The process initiates with the uptake of oxLDL by macrophages, leading to the transformation of these cells into foam cells. The hallmark markers associated with foam cell development include scavenger receptors such as CD36 and SR-A, which facilitate the internalization of modified lipoproteins. Additionally, acyl-coenzyme A:cholesterol acyltransferase (ACAT) and lipid droplet-associated proteins contribute to lipid accumulation within the cells.

In PWH on ART and presenting a low-to-moderate traditional atherosclerotic cardiovascular disease (ASCVD) risk, but with an abundance of plaque, elevated levels of IL-18 and IL-1β were found to be connected to the Leaman score. The Leaman score serves as an indicator of atherosclerotic burden, which is associated with cardiovascular events in the general population [1,13]. The same study found a correlation between the NLRP3 inflammasome and coronary artery plaque composition and severity in PWH on ART. In a collaborative study [14], we showed that PWH who have not received ART compared to uninfected controls have higher levels of plasma caspase-1, a component of the NLRP3 inflammasome. 

Within human carotid atherosclerotic plaques, components of the NLRP3 inflammasome are mainly expressed in macrophages and foam cells, and only sporadically in smooth muscle cells [15]. Consequently, the role of NLRP3 inflammasome activation in the pathogenesis of atherosclerosis is studied in monocytes and macrophages, which are abundantly present in the developing atherosclerotic lesion.

REPRIEVE, The Randomized Trial to Prevent Vascular Events in HIV, is the first large-scale randomized clinical research trial to test a strategy for cardiovascular prevention in PWH. Recent results from REPRIEVE showed that PWH who received pitavastatin, a lipid-lowering drug, reduced major heart disease events by 35% and reduced major heart disease events or death from any cause by 21% compared to placebo after 5 years. These results show that blocking lipid uptake and accumulation in PWH lower the cardiovascular risk. In addition, there were no differences in baseline LDL levels, and a reduction in immune activation and inflammation by pitavastatin may also have influenced the outcomes [16]. 

The atherogenic role of the NLRP3 inflammatory trigger in HIV infection has not been studied in detail. Awareness of the mechanism of the inflammatory complex and its involvement in HIV atherosclerosis can help explain what triggers this pathway. Here, we sought to elucidate the role of NLRP3 inflammasome in the foam cell formation process, one of the hallmarks of HIV-associated atherogenesis.

## 2. Results

### 2.1. HIV Increases Foam Cell Formation and NLRP3 Downstream Cytokines

**Figure 1:**
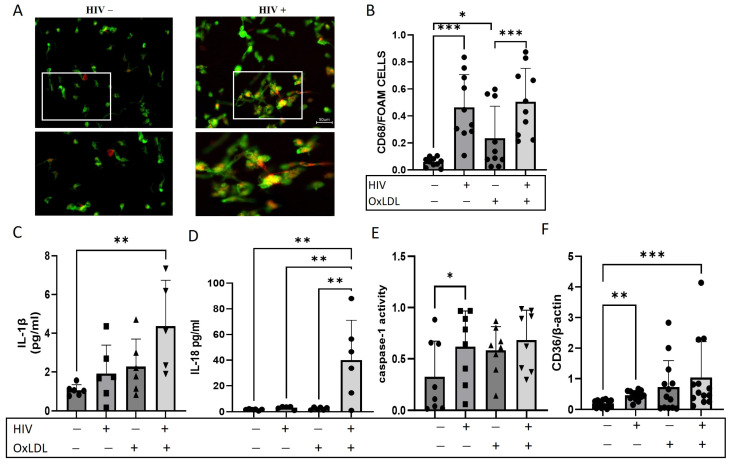
Foam cell formation in HIV-infected macrophages. (**A**) Representative images of Oil red O staining of monocyte-derived macrophages (MDM) cells isolated from PBMCs of healthy donors after infected with HIV and/or treated with oxLDL (100 μg/mL) for 24 h. Cells were stained with Oil red O (red) for lipids and CD68 (green) for macrophages. Scale bar 50 μM. The white square box represents the enlarged area shown in the bottom images (**B**) Quantification of Oil red O staining is shown where each point on the graph corresponds to the average value calculated from five different images captured for each sample. Double-positive cells for macrophage marker CD68 and Oil Red O for lipids are considered foam cells. IL-1β (**C**) and IL-18 (**D**) measured by ELISA in cell culture supernatant. (**E**) Caspase-1 activity measured in cells was calculated based on number of positive green cells following manufacturer’s instruction, and (**F**) CD36 RNA expression was measured by qPCR. * *p* < 0.05, ** *p* < 0.01, *** *p* < 0.001. All data are presented as mean ± SEM. − represents the absence of a treatment, + represents the presence of a treatment.

Macrophage foam cells play a key role in the development of atherosclerosis. Here, the ability of HIV to induce foam cell formation, a hallmark of atherosclerosis, was examined. Monocyte-derived macrophages (MDM) isolated from PBMCs of healthy donors were infected with HIV-ADA, a macrophage-tropic strain of HIV. After 4 days in culture, MDMs were incubated overnight with oxLDL to stimulate the formation of foam cells. (see Supplementary Materials Figure S1 for experimental timelines) CD68^+^/Oil Red O^+^-positive cells were considered foam cells. Both uninfected and no oxLDL and single conditions were used as controls (Figure 1). Successful HIV infection of MDMs was assessed by measuring p24 in cell supernatant by ELISA (Supplementary Materials Figure S2).

HIV infection increased foam cell formation compared to HIV−negative monocytes (*p* < 0.0004). There was no further increase in foam cell formation when oxLDL was added to the HIV–infected cells (Figure 1A,B). As expected, the oxLDL alone increased the percentage of foam cells (*p* < 0.04) compared to untreated macrophages, and HIV plus oxLDL significantly elevated the percentage of foam cells compared to oxLDL alone (*p* < 0.0008). IL-1β and IL-18 in the supernatant and caspase-1 activity in MDMs were measured (Figure 1C–E). Both IL-1β and IL-18 were increased in the cell culture supernatant after HIV infection and oxLDL treatment compared to the uninfected controls (Figure 1C; *p* < 0.005 and Figure 1D; *p* < 0.002, respectively). Caspase-1 activity was significantly increased with HIV as well compared to controls (Figure 1E, *p* < 0.02). 

To further explore the effect of HIV on foam cell formation, the expression of the scavenger receptor CD36 known to promote inflammation and foam cell formation was measured in MDMs. HIV increased the expression of CD36 compared to the HIV−negative control (Figure 1F, *p* < 0.008), and the addition of oxLDL enhanced CD36 expression (Figure 1F, *p* < 0.0002, HIV+ oxLDL vs. untreated macrophages).

To determine a possible effect of HIV proteins on foam cell formation, MDMs were treated with HIV proteins: tat, nef and gp120. No significant effect was seen with any of the HIV proteins examined on foam cell formation (Supplementary Materials Figure S3A–C). Taken together, our results show that HIV increased foam cell formation and the expression of IL-1β and IL-18 secretion, and caspase-1 and CD36 levels in macrophages, which are abundantly present in the developing atherosclerotic lesion.

### 2.2. Transcriptomic and Pathway Changes in Macrophages after HIV Infection and oxLDL Treatment

**Figure 2:**
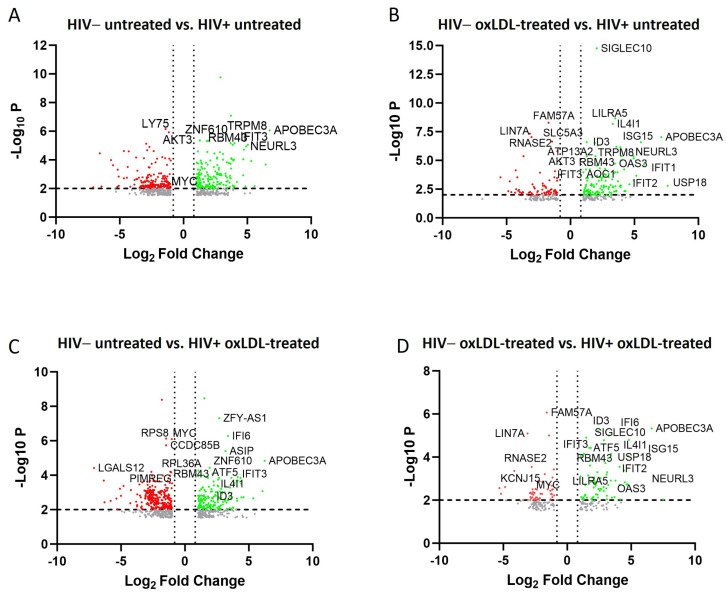
Volcano plot of differentially expressed genes. The Volcano plots show the most significantly expressed genes found by univariate analysis (cutoff value 0.02). The plots represent significantly different genes in treated PBMCs in the 2 groups. HIV− untreated vs. HIV+ untreated (**A**), HIV− oxLDL treated vs. HIV+ untreated (**B**), HIV− untreated vs. HIV+ oxLDL treated (**C**) and HIV− oxLDL treated vs. HIV+ oxLDL treated (**D**). The scatterplot of the negative log10-transformed *p*-values plotted against the log2 fold change, where negative values indicate downregulated genes (in red), while positive values reflect upregulated genes (in green). Genes with large fold change values lie far from the vertical threshold line at log2 fold change = 0, indicating whether the genes are up or downregulated.

To further investigate the global transcriptomic changes after HIV infection and oxLDL treatments, RNAseq analysis of MDM was performed on four groups; HIV−, HIV+, HIV− oxLDL+, and HIV+ oxLDL+. More than 800 genes (Supplementary Materials Table S1) were differentially expressed in the HIV-infected, and HIV-infected plus oxLDL group compared to the control group, based on a cutoff value of fold change > |2| and FDR < 0.01. Some of the key genes are shown in the volcano plots (Figure 2A).

Denoted DEGs from expression comparison were evaluated among HIV−, HIV− plus oxLDL, HIV+, and HIV+ plus oxLDL groups by transcripts per million (TPM), and the statistically significant DEGs are shown in Supplementary Materials Figure S4. For comparison between HIV+ and HIV+ plus oxLDL and HIV− and HIV− plus oxLDL, there were just one and zero differentially expressed genes, respectively. Here, we found that the expression of genes involved in lipid metabolism and atherosclerosis is increased after HIV infection and oxLDL and by oxLDL treatment itself. The genes involved are: IFIT1, IFIT2 IFIT3, NEURL3, ID3, ISG15, TNFS18, RBM43, and TRPM8. Principle component analysis (PCA) showed clustering of animals from the analysis (Supplementary Materials Figure S5), where HIV+ and HIV+ plus oxLDL samples clustered similarly, while HIV− samples were distinct from both HIV+ and HIV+ plus oxLDL transcriptional profiles. Taken together, changes at the transcriptomic level and pathway activation further shed light on the pathogenic role of HIV and inflammasome in HIV-associated atherogenesis.

### 2.3. Foam Cell Formation Is Reduced after HIV Infection and NLRP3 Inhibition

**Figure 3:**
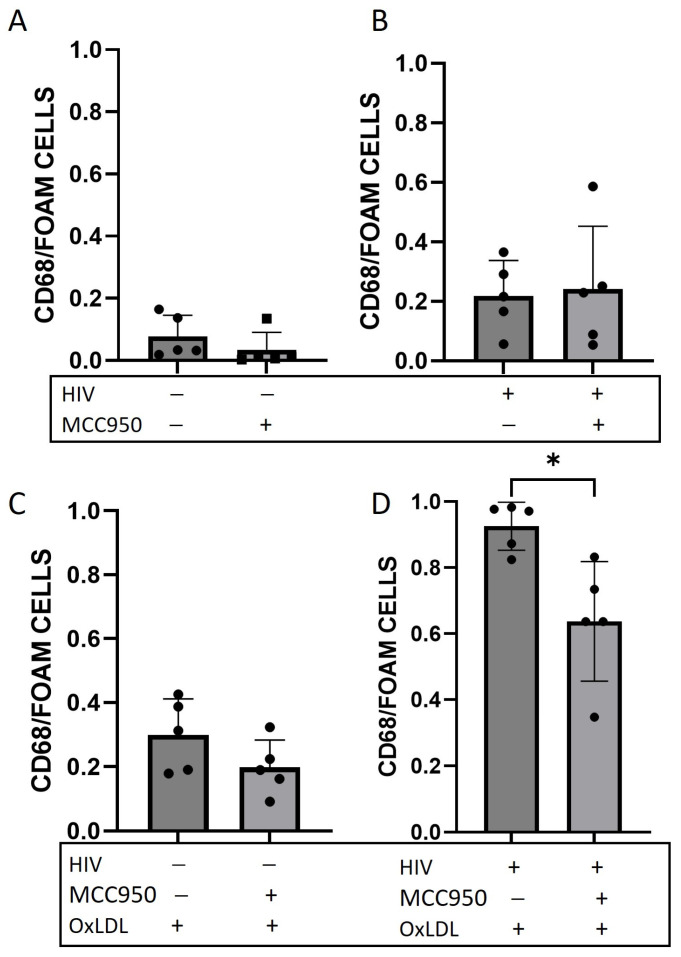
Foam cell formation in HIV-infected macrophages and inhibition with NLRP3 inhibitor MCC950. Quantification of monocyte-derived macrophages (MDM) cells isolated from PBMCs of healthy donors either HIV-infected (HIV+) or uninfected (HIV−) and/or oxLDL-treated (oxLDL+) or non-treated (oxLDL−) and/or treated with NLRP3 inhibitor MCC950 (100 μg/mL) for 24 h. HIV− vs. HIV− MCC950 treated (**A**), HIV+ vs. HIV+ MCC950 treated (**B**), HIV− oxLDL treated vs. HIV− MCC950 and oxLDL treated (**C**), HIV+ oxLDL treated vs. HIV+ MCC950 and oxLDL treated (**D**). * *p* < 0.05. All data are presented as mean ± SEM. − represents the absence of a treatment, + represents the presence of a treatment.

To further analyze the effect of NLRP3, the same protocol was used for infection and oxLDL treatment, but MDMs were treated with a potent NLRP3 inhibitor, MCC950 (0.4 μg/mL) (see Supplementary Materials Figure S1 for experimental outline, Figure 3A–D). As described above, foam cells formed after HIV infection and oxLDL treatment. Foam cell formation only decreased after treatment with MCC950 in the HIV infection and oxLDL treatment condition (Figure 3D), and not in uninfected (Figure 3A), or only HIV infected (Figure 3B) or oxLDL treated (Figure 3C). The only significant decrease in foam cells after MCC950 was in the HIV-infected and oxLDL treatments, which might be due to the high baseline foam cell formation with HIV and oxLDL. These results show that foam cell formation is significantly decreased when NLRP3 is inhibited, demonstrating the critical involvement of NLRP3 in foam cell development in HIV-mediated atherogenesis.

### 2.4. ART and HIV Increase Foam Cell Formation

**Figure 4:**
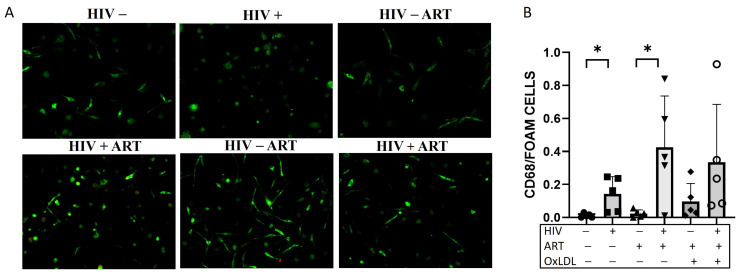
Foam cell formation in HIV-infected ART-treated macrophages. (**A**) Representative images of Oil red O staining of monocyte-derived macrophages (MDM) cells isolated from PBMCs of healthy donors that were HIV-infected (HIV+) or uninfected (HIV−) and/or ART-treated (ART+), and/or oxLDL-treated (oxLDL+) or non-treated (oxLDL−). Cells were stained with Oil red O (red) for lipids and CD68 (green) for MDM. (**B**) Quantification of Oil red O staining. * *p* < 0.05. All data are presented as mean ± SEM. − represent the absence of a treatment, + represent the presence of a treatment.

ART is known to increase endothelial dysfunction in the short-term partially due to endothelial activation [3]. There is clinical evidence that PWH on ART still have accelerated atherosclerosis. Here, we examined if treatment with a clinically relevant ART regimen would affect foam cell formation, as shown in Figure 4. 

A combination of antiretroviral drugs (tenofovir (1 μg/mL), emtricitabine (2 μg/mL), and raltegravir (0.1 μg/mL), were used to treat cells for 6 days after infection followed by oxLDL treatment. In line with previous experiments, we confirmed an increase in foam cell formation after HIV infection. ART during HIV infection increased foam cell formation compared to ART treatment itself. However, we did not detect any statistically significant changes in the expression of NLRP3 inflammasome components with ART.

### 2.5. PWH Show an Increased Foam Cell Formation and NLRP3 Expression 

**Figure 5:**
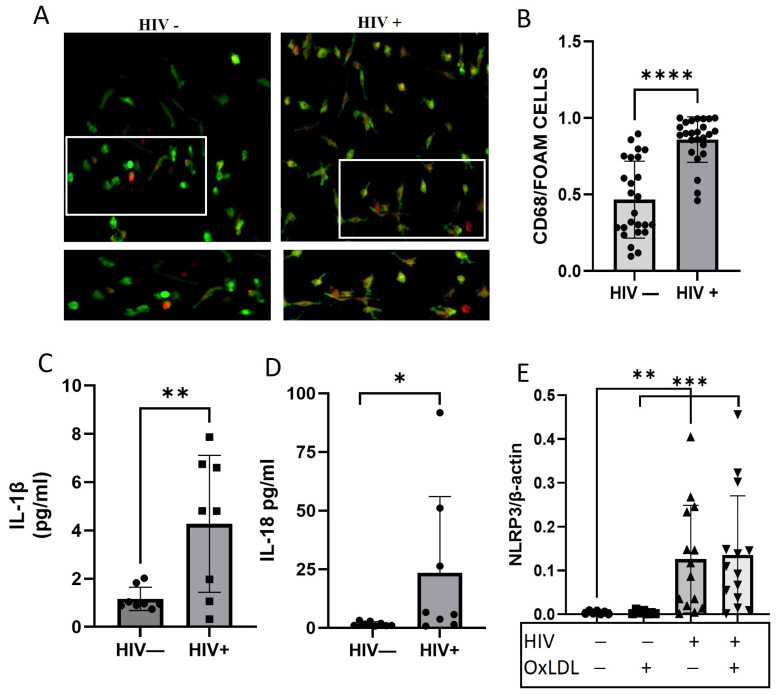
Foam cell formation is elevated in PWH. (**A**) Representative images of Oil red O staining of monocyte-derived macrophages (MDM) cells isolated from PBMCs of PWH (HIV+) or healthy donors (HIV−) and treated with oxLDL. Cells were stained with Oil red O (red) for lipids and CD68 (green) for MDM. The white square box represents the enlarged area shown in the bottom images. (**B**) Quantification of Oil red O staining. IL-1β (**C**) and IL-18 (**D**) measured by ELISA in cell culture supernatant. (**E**) NLRP3 RNA expression measured by qPCR. * *p* < 0.05, ** *p* < 0.01, *** *p* < 0.001, **** *p* < 0.0001. All data are presented as mean ± SEM. − represent the absence of a treatments, + represent the presence of a treatment.

Next, we examined if monocytes from PWH had enhanced ability to become foam cells compared to those from matched HIV− controls. PBMCs from 25 PWH and 25 HIV−matched controls (Supplementary Materials Table S2) were used. MDMs from PWH have an increased ability to form foam cells compared to healthy donors (Figure 5A,B; *p* < 0.0001). 

In the supernatant, the downstream cytokines, IL-1β, and IL-18 (Figure 5C,D) were also increased in PWH compared to controls. NLRP3 is highly expressed basally in PWH and with oxLDL compared to controls. OxLDL did not significantly increase the level of NLRP3 compared to HIV alone, suggesting HIV itself is atherogenic. The expression of the other NLRP3 inflammasome components, caspase-1, adaptor molecule apoptosis-associated speck-like protein containing a CARD (ASC), IL-18 and IL-1β, and IL-1β precursor, was tested by qRT-PCR, and an increased expression of IL-1β precursor (Supplementary Materials Figure S6D), but a decreased expression of ASC (Supplementary Materials Figure S6B) was seen. Taken together, our results show that HIV increased foam cell formation and the expression of NLRP3 downstream cytokines IL-1β, IL-18, and NLRP3, which are abundant in pro-atherosclerotic lesion.

## 3. Discussion

The present study aimed to investigate the potential role of the NLRP3 inflammasome in HIV-mediated atherogenesis and foam cell formation using an in vitro HIV infection model and a cohort of PWH. Our previous study [17] demonstrated that global caspase-1 knockout decreases plaque size in the thoracic region of the aorta in Tg26+/−/ApoE−/− /Casp-1−/− mice and foam cell formation, and, moreover, the deficiency of caspase-1 in immune cells attenuates atherogenesis in HIV transgenic mice. A study investigated the potential of monocytes to promote atherosclerosis in a cohort of 39 PWH on ART with no signs of CVD, and 25 HIV-uninfected controls of comparable age, sex, smoking status, and CVD risk. They found that monocytes from PWH have increased potential to form foam cells compared with controls in an ex vivo experiment. Foam cell formation was linked to an impaired ability of monocytes to undergo reverse transmigration, and a decreased ability to efflux cholesterol ex vivo. Importantly, foam cell formation decreased with the duration of viral suppression [18].

Here, we indicate that HIV infection contributes to increased foam cell formation and the expression of NLRP3 inflammasome downstream cytokines IL-1β and IL-18. These data were confirmed in monocytes from PWH with increased foam cell formation, IL-1β, IL-18, and NLRP3 compared to HIV− controls. HIV proteins, such as tat, gp120, and nef, did not have any effect on foam cell formation. There are limitations to our studies from clinical samples, which include unpaired demographics of the PWH and HIV− donors. Although matched on sex, we were unable to match the PBMCs on race and age. The PWH had more African American people (68% vs. 16%) and the HIV− donors group had more Caucasian people (76% vs. 24%) (*p* = 0.012, chi-square). The age was higher in PWH than controls (51.72 vs. 46.25, Mann–Whitney *t* test *p* = 0.007) (Supplementary Materials Table S2). In addition, although all HIV− donors had no hypertension, were non-smokers, and were negative for HBV, HCV, and CMV, we did not have matching information on immune parameters, diabetes, lipids, and other viral antibody titers.

Foam cell formation is a hallmark of atherosclerosis, where macrophages internalize modified LDL, leading to the accumulation of lipids and the formation of foam cells within the arterial intima. HIV infection itself acts as a trigger for inflammasome activation [6]. This study demonstrated that HIV infection increased foam cell formation. Moreover, the increased expression of the scavenger receptor CD36 in response to HIV and oxLDL, suggests a mechanism by which these factors contribute to atherogenesis. The findings are in line with previous studies showing that oxLDL and cholesterol deposition are potent inflammatory molecules linked to vascular inflammation. Additionally, caspase-1 activity, a key mediator of inflammasome activation, was found to be elevated in HIV-infected PBMCs treated with oxLDL, further implicating the inflammasome in HIV-associated inflammation. To further demonstrate the specific effect of the NLRP3 inflammasome, we used a potent NLRP3 inhibitor, MCC950. MCC950 reduced foam cell formation, highlighting the critical involvement of NLRP3 in this process. 

Our bulk RNA sequencing results showed upregulated RNAs expression of genes involved in lipid metabolism and atherosclerosis after HIV infection and oxLDL treatment. A previous study identified IFIT1, IFIT2, IFIT3, and ISG15 as immune-related hub genes of atherosclerosis [19]. IFIT2 expression is increased in human monocyte-derived dendritic cells by HIV-1 Vpr. ISG15, first described as an antiviral molecule against many viruses, participates in numerous cellular processes, from immune modulation to the regulation of genome stability. ISG15 is found to regulate lipid metabolism in bone marrow-derived macrophages. Another study highlighted how the absence of ISG15 impacts macrophage lipid metabolism in the context of viral infections [19]. Several studies have demonstrated its relevance in the control of cholesterol storage, the attenuation of lipid-induced ER stress, and the restriction of atherosclerosis development through the regulation of CE levels, and, therefore, the cellular LD content. NEURL3 participates in the accumulation of intracellular cholesterol [20]. Jing G et al. [21] showed that TNFSF18 expression, which promoted p-STAT1 phosphorylation to activate the proteins VCAM1, ICAM1, ITGAD, and ITGB3, exacerbates coronary microcirculation disorder in an atherosclerotic mouse model. Also, upregulation of RBM43 shows the potential diagnostic and prognostic value for the occurrence of ischemic events. TRPM8 is expressed in cells of the monocyte/macrophage lineage in humans. A study has shown an essential role for TRPM8 in the regulation of human monocyte/macrophage differentiation and function [22]. A loss of ID3 accelerates atherosclerosis development in ApoE-depleted mice fed an atherogenic diet [23], but here we found that the expression of genes involved in the lipid metabolism and atherosclerosis are increased after HIV infection and oxLDL treatment itself.

Increased risk of ASCVD has been observed among PWH due to heightened systemic immune activation and inflammation [24]. PWH before ART show a higher level of caspase-1 compared to people without HIV. ART decreases caspase-1 levels but they still tend to be higher than people without HIV, indicating that there is still inflammation and activation of NLRP3 inflammasome [14]. Combination ART use is associated with a 26% relative increase in the rate of myocardial infarction (MI) per year of exposure to ART [25]. A study demonstrated that ART increases pro-atherogenic small LDL particles and VLDL [26]. ART is also known to increase endothelial dysfunction in the short-term, partially due to endothelial marker activation [3]. Furthermore, our study examined the impact of ART on foam cell formation. The findings indicated that ART increased foam cell formation after HIV infection, reinforcing the complex relationship between HIV, ART, and atherogenesis. These observations emphasize the need for a comprehensive understanding of the effects of ART on cardiovascular health in PWH.

## 4. Materials and Methods

### 4.1. Study Participants

Twenty-five cryopreserved PBMCs were obtained from both men and women with HIV from the Clinical and Translational Research Support Core (CTRSC) of the Comprehensive NeuroHIV Center (CNHC), (NIH P30 MH092177 (Kamel Khalili, PI; Brian Wigdahl, CTRSC Director). Participant samples were collected under IRB protocol 1609004807 held at Drexel University (Brian Wigdahl, PI). Twenty-five cryopreserved PBMCs from HIV-seronegative control men and women were acquired from BioIVT (Westbury, NY, USA), with an age range of 27–71 (average 46.52). PBMCs were only obtained from people without a history or symptoms of heart disease and without hypertension as a comorbidity. Other than HIV disease, inclusion and exclusion criteria were identical for groups of samples. The PBMCs were from PWH age 44–62 years (average 51.72) without prior history of cardiac disease or symptoms suggestive of cardiac disease. HIV-infected and non-HIV control participants with known renal disease or estimated creatinine clearance <60 mL/min were excluded. All PWH were receiving ART at the time of the blood draw and had received stable therapy for >3 months. The PWH had more African American people (68% vs. 16%) and the HIV− donors group had more Caucasian people (76% vs. 24%) (*p* = 0.012, chi-square). The age was higher in PWH than controls (51.72 vs. 46.25, Mann–Whitney *t* test *p* = 0.007) (Supplementary Materials Table S2).

### 4.2. ELISA

Cell culture supernatant was used to perform all ELISAs. Human IL-18 ELISA kit (Abcam, Cambridge, UK), human IL-1 beta/IL-1F2 Quantikine HS ELISA kit (R&D system, Minneapolis, MN, USA) and HIV p24 (ABLinc., Rockville, MD, USA) were utilized following the manufacturer’s recommendations.

### 4.3. FLICA-FAM Caspase-1 Staining 

Active caspase-1 activity in macrophages was determined by FAM FLICA caspase-1 assay kit (ImmunoChemistry Technologies, Davis, CA, USA). All procedures were performed following the manufacturer’s instructions. Residual red blood cells (PBC) in PBMCs were lysed using RBC lysis buffer for 10 min at room temperature. After washing with 1 × PBS, cells were then stained with caspase-1 inhibitor FAM-YVAD-FMK at 37 °C for 1 h, followed by washing with 1× apoptosis wash buffer provided in the kit. In the end, the supernatants were discarded, and the cells were resuspended with 300 μL of 1× apoptosis wash buffer. Cells were also stained using an anti-CD68 antibody (Novus Biologicals, Centennial, CO, USA), a macrophage marker. 

### 4.4. Foam Cell Formation Assay

PBMCs were thawed and monocytes were isolated using EasySep™ human monocytes isolation kit (STEMCELL Technologies Inc., Vancouver, BC, Canada), plated, and differentiated into macrophages for 8 days. M-CSF was used to stimulate MDM at a concentration of 50 ng/mL (STEMCELL Technologies Inc., Vancouver, BC, Canada). To induce foam cell formation, adherent macrophages were treated with 100 μg/mL oxLDL (Kalen Biomedical, Montgomery Village, MD, USA) for 24 h then double-stained with 0.4% oil red O for detection of lipids and anti-CD68 antibody for detection of macrophages. Double-stained CD68^+^/Oil Red O^+^-positive cells are considered foam cells. The percentage of macrophages that were also positive for oil red O was quantified in five 20× fields. Each point on the graph corresponds to the average value calculated from five different images captured for each sample. Antiretroviral drugs used were tenofovir (1 μg/mL), emtricitabine (2 μg/mL), and raltegravir (0.1 μg/mL) (Cayman chemical company, Ann Arbor, MI, USA). HIV tat, nef, and gp120 were used at a concentration of 100 ng/mL. Macrophage tropic HIV-ADA was used to infect MDMs at a concentration of 10 ng/mL of p24, a kind gift from Dr. Peter Gaskill (Drexel University School of Medicine, Philadelphia, PA, USA).

### 4.5. qRT-PCR

Total RNA was extracted from cells using Monarch^®^ Total RNA Miniprep Kit (New England Biolabs, Ipswich, MA, USA) according to the manufacturer’s instructions. An amount of 100 ng of RNA was used for each reaction using Luna^®^ Universal Probe One-Step RT-qPCR Kit (New England Biolabs, Ipswich, MA, USA) using Roche light cycler 96 (Roche, Indianapolis, IN, USA). The reaction conditions were as follows: reverse transcription 55 °C 10 min, initial denaturation 95 °C 1 min, denaturation 95 °C 10 s, extension 60 °C 30 s, 45 cycles. Data for relative expression were presented using the 2^−ΔΔCt^ method. 

### 4.6. RNA Extraction, Library Preparation, Sequencing, and Analysis

Total RNA was extracted using RNeasy mini kit (Qiagen, Valencia, CA, USA) from MDMs, and RNA samples first underwent a quality control (QC) assessment using the Agilent Bioanalyzer (Agilent, Santa Clara, CA, USA) and submitted for sequencing samples that had an RNA Integrity Number (RIN) >8. cDNA library was processed using Illumina HiSeq 2 × 150 bp sequencing (Genewiz/Azenta). The fragments were mapped to the ensemble homo sapiens reference genome GRCh38.p14 using the STAR aligner [27]; then, gene expression and nucleotide variation were evaluated as previously described [28,29]. The QC and alignment were performed using nf-core/rnaseq pipeline, then the results were fed into Partek Flow where DEseq2 was used for differential analysis. Differentially expressed genes (DEGs) were determined as genes that had a fold change (FC) greater than |2.0| and had a false discovery rate (FDR) less than 0.01. DEGs were then used for graphical comparisons and ingenuity pathway analysis (IPA). To eliminate mapping errors and reduce potential mapping ambiguity due to homologous sequences, several filtering steps were applied. Specifically, we required [1] the mapping quality score of each read to be ≥30, [2] reads from the same pair were mapped to the same chromosome with expected orientations and the mapping distance between the read pair was <500,000 bp, and [3] each read was uniquely mapped to the genome. All subsequent analyses were based on filtered aligned reads.

### 4.7. Statistics

The data were analyzed using GraphPad Prism 9.0 software (La Jolla, CA, USA) and presented as the mean ± the standard error of the mean (SEM). Experiments were performed using at least two biologically distinct replicates. Outliers were analyzed using the ROUT method and the values 3-fold greater than the SEM were excluded. D’agostino–Pearson omnibus normality test, Anderson–Darling test, Shapiro–Wilk test, and Kolmogorov–Smirnov normality test with Dallal–Wilkinson–Lilliefor *p*-value were used to test for normality and lognormality. One-way ANOVA with Bonferroni correction was used for multiple comparisons, and Dunn’s test was used as a post-hoc for multiple comparison test. For data not normally distributed, the Mann–Whitney test was used. Significant differences were determined at *p* < 0.05.

## 5. Conclusions

The findings of this study contribute to a better understanding of the complex interplay between HIV infection, inflammation, and cardiovascular disease, suggesting potential targets for therapeutic interventions aimed at reducing the risk of atherosclerosis in this population. Further research is needed to elucidate the intricate interactions between immune responses, lipid metabolism, and cardiovascular health in PWH, with the goal of improving clinical outcomes and quality of life for PWH.

## Data Availability

Authors will make all materials used to conduct this research available to other researcher upon a reasonable request.

## Supplementary Materials

The following supporting information can be downloaded at: https://www.mdpi.com/article/10.3390/ijms25042367/s1.
