# Supplementary material for: HIV Infection Drives Foam Cell Formation via NLRP3 Inflammasome Activation
Source: Int J Mol Sci. 2024 Feb 17;25(4):2367. doi: 10.3390/ijms25042367 (PMC10889596; doi:10.3390/ijms25042367)
Supplement: Supplementary file 1 [file ijms-25-02367-s001.zip › ijms-2832891-supplementary.pdf]

Supplemental material

HIV infection drives foam cell formation via NLRP3 inflammasome activation

Maurizio Caocci [1], Meng Niu [2],

Howard S. Fox [3] and Tricia H. Burdo [1*]

[1] Department of Microbiology, Immunology, and Inflammation; Center for Neurovirology and Gene Editing; Lewis Katz School of Medicine at Temple University, Philadelphia, PA 19140, USA

[2]Department of Genetics, Cell Biology and Anatomy; and Bioinformatics and Systems Biology Core, University of Nebraska Medical Center, Omaha, NE 68198, USA.

[3]Department of Neurological Sciences, University of Nebraska Medical Center, Omaha, NE 68198, USA.

*Corresponding author: Tricia H. Burdo, Department of Microbiology, Immunology, and Inflammation; Center for Neurovirology and Gene Editing; 3500 N Broad St. MERB 760, Lewis Katz School of Medicine at Temple University, Philadelphia, PA 19140, USA. Email: burdot@temple.edu

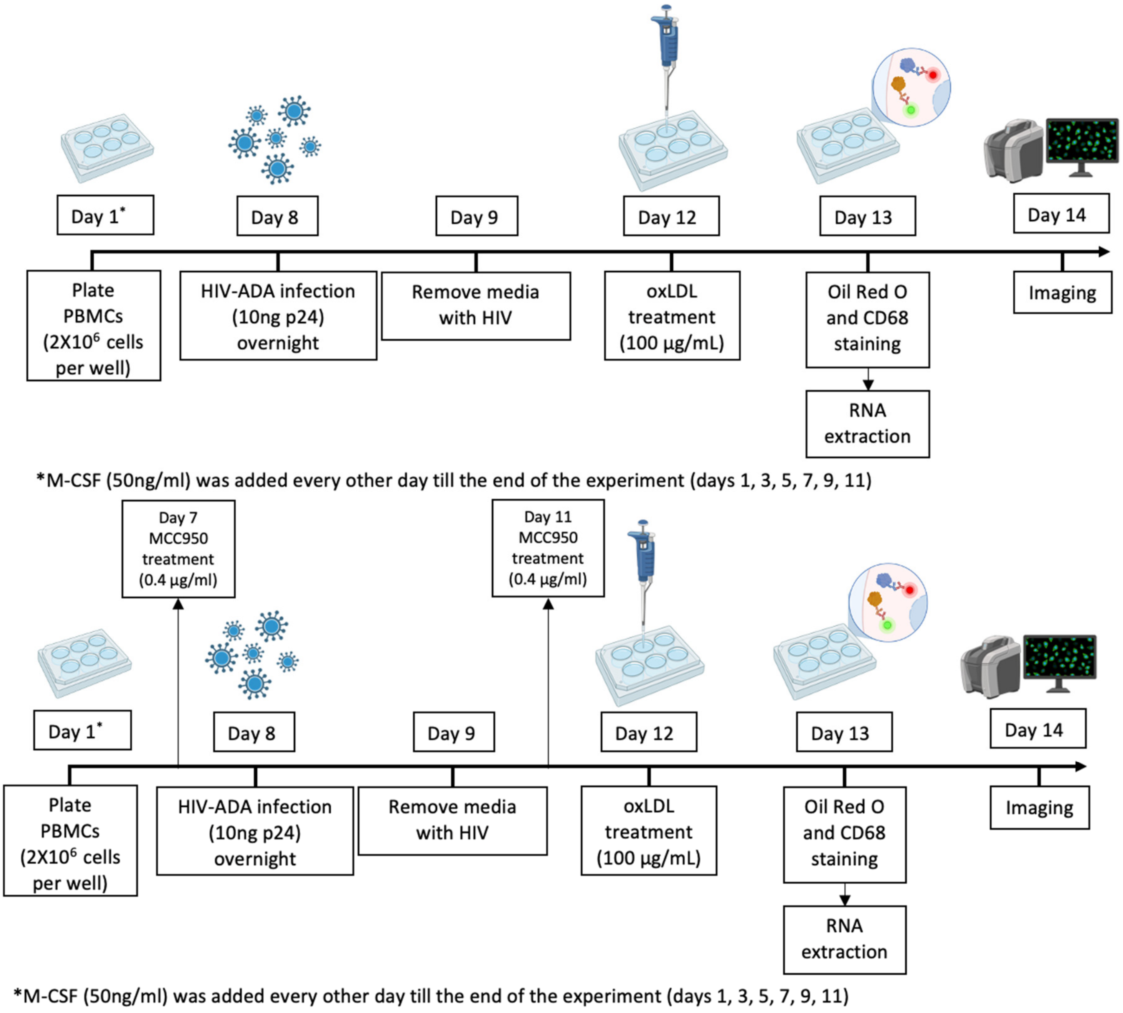

Supplemental Figure S1. Experimental set up and timeline.

Timeline indicating HIV infection, treatments, and staining protocol. The figure was partially made using Biorender. The top graphic relates to the data from Figure 1 and the bottom graphic is the experimental set-up for Figure 3.

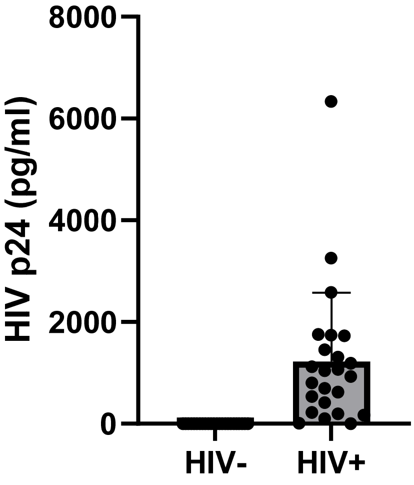

Supplemental Figure S2. HIV p24 measured by ELISA nine days after infection with HIV-ADA.

Expression of HIV p24 measured by ELISA in HIV uninfected and HIV infected samples after nine days of infection.

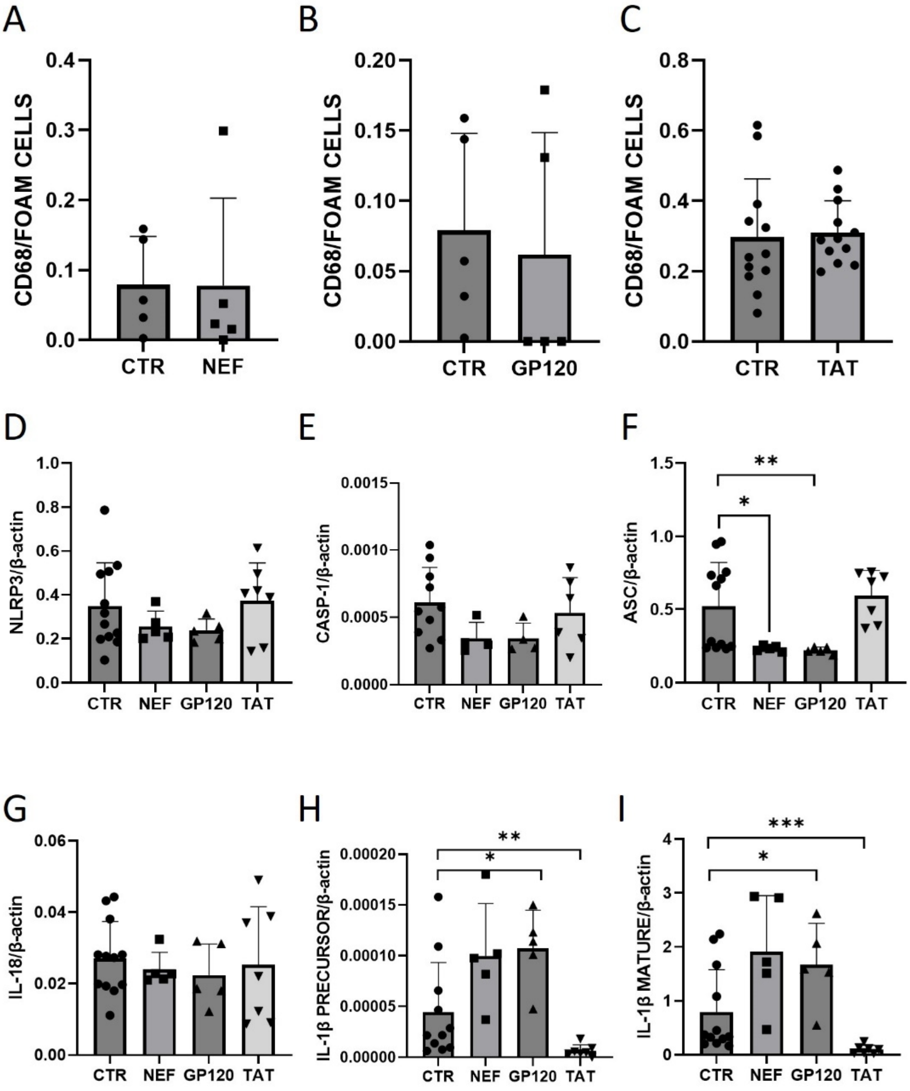

Supplemental Figure S3. Foam cell formation and expression of NLRP3 inflammasome after HIV Tat, Gp120, and Nef treatment

Oil red O staining of monocyte derived macrophages (MDM) cells isolated from PBMCs of healthy donors cultured for 8 days, treated/untreated with HIV nef (A), HIV gp120 (B), HIV tat (C), and treated with/without oxLDL (100 μg/mL) for 24 hours. Cells were stained with Oil red O (red) for lipids and CD68 (green) for MDM. Expression of NLRP3 (A) caspase-1 (B), ASC (C), IL-18 (D), IL-1β precursor (H) and mature form (I) measured by qPCR.

Supplemental Table S1. Summary of Differentially Expressed Genes

Comparison	Upregulated Genes	Downregulated Genes	Total Significantly Differentially Expressed Genes
HIV- untreated vs. HIV+ untreated	365	470	835
HIV- oxLDL-treated vs. HIV+ untreated	232	218	450
HIV- untreated vs. HIV+ oxLDL-treated	341	515	856
HIV- oxLDL-treated vs. HIV+ oxLDL-treated	163	204	367

HIV-: uninfected, HIV+: HIV-infected

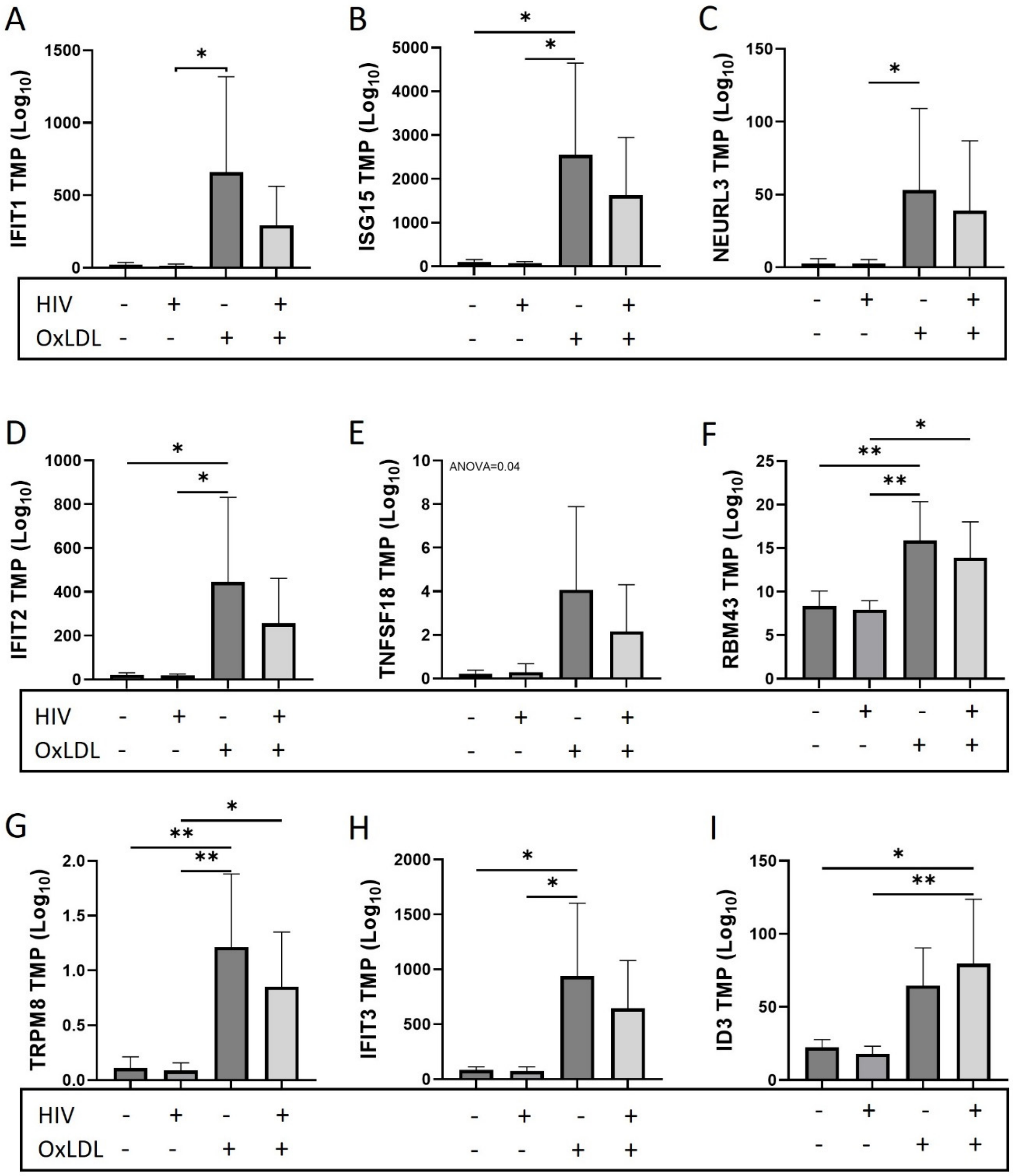

Supplemental Figure S4. Comparison of Significant DEGs from HIV-, HIV+, HIV- oxLDL, HIV+ oxLDL Groups

Significant DEGs identified from comparisons among the four groups by transcripts per million (TPM, log transformed base 10). DEG TPMs were compared by Kruskal Wallis test and post-hoc Dunn's multiple comparison.

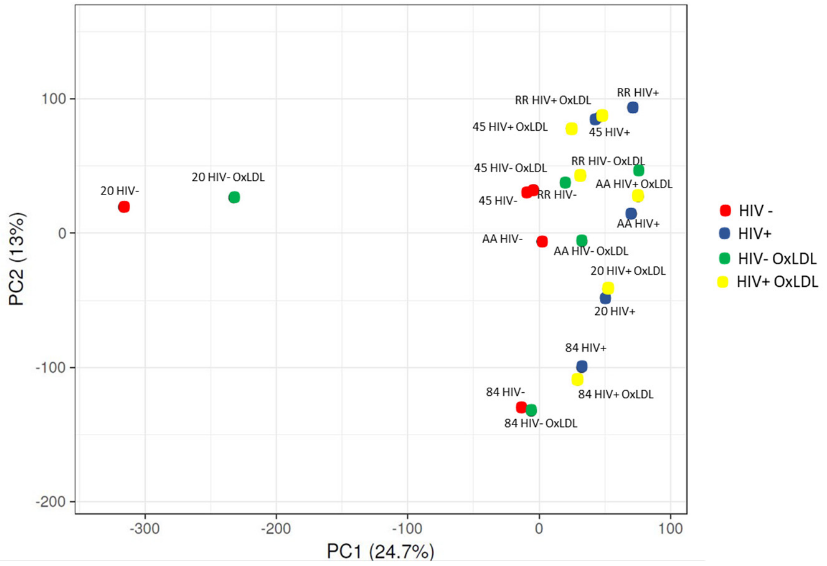

Supplemental Figure S5. Principal Component Analysis (PCA) Plot

PCA plot shows distribution of mapped genes from HIV-, HIV+, HIV- oxLDL, HIV+ oxLDL comparison.

Supplemental Table 2. PWH and HIV- donor demographics

Characteristics	PWH (N=25)	Controls (N=25)
Age – years*	51.72 (44-62)	46.52 (27-71)
Female sex (%)	36	36
Male sex (%)	64	64
Race – (%)**		
African American	68	16
Caucasian	24	76
Hawaiian	4	0
Multiracial	4	0
Asian	0	4
Hispanic	0	4
Currently on ART (%)	100	N/A
Average years on ART	19.05 (5.31-33.98)	N/A
Undetectable PVL (%)	92	N/A
Detectable PVL (%)	8	N/A
CD4+ count (cells/uL)	653.96 (213-1266)	N/A
CD8+ count (cells/uL)	1149 (667-1981)	N/A
HBV+ (%)	8	0
HCV+ (%)	32	0
CMV+ (%)	0	0
HPV+ (%)	24	N/A
HSV-1+ (%)	20	N/A
HSV-2 (%)	20	N/A
TB+ (%)	12	N/A
Diabetes+ (%)	24	N/A
Smoking status	Non-smokers	Non-smokers
Elevated lipids (%)	36	N/A

Age, Average years on ART, CD4 and CD8 counts are all mean (range). All other data is percentage (%). PWH= people with HIV, N/A= not available, HBV= hepatitis B virus, HCV= hepatitis C virus, CMV= cytomegalovirus, HPV= human papillomavirus, HSV= herpes simplex virus, TB= tuberculosis, PVL= plasma viral load. Undetectable viral loads were all less than 30 copies/mL. Only 2 PWH had detectable viral loads of 153 and 69975 copies/mL. All co-infections are based on medical history and past serology and not current active infections. There was significant differences in age and race of the groups. * Mann Whitney t test p=0.007, **chi-square test p=0.012.

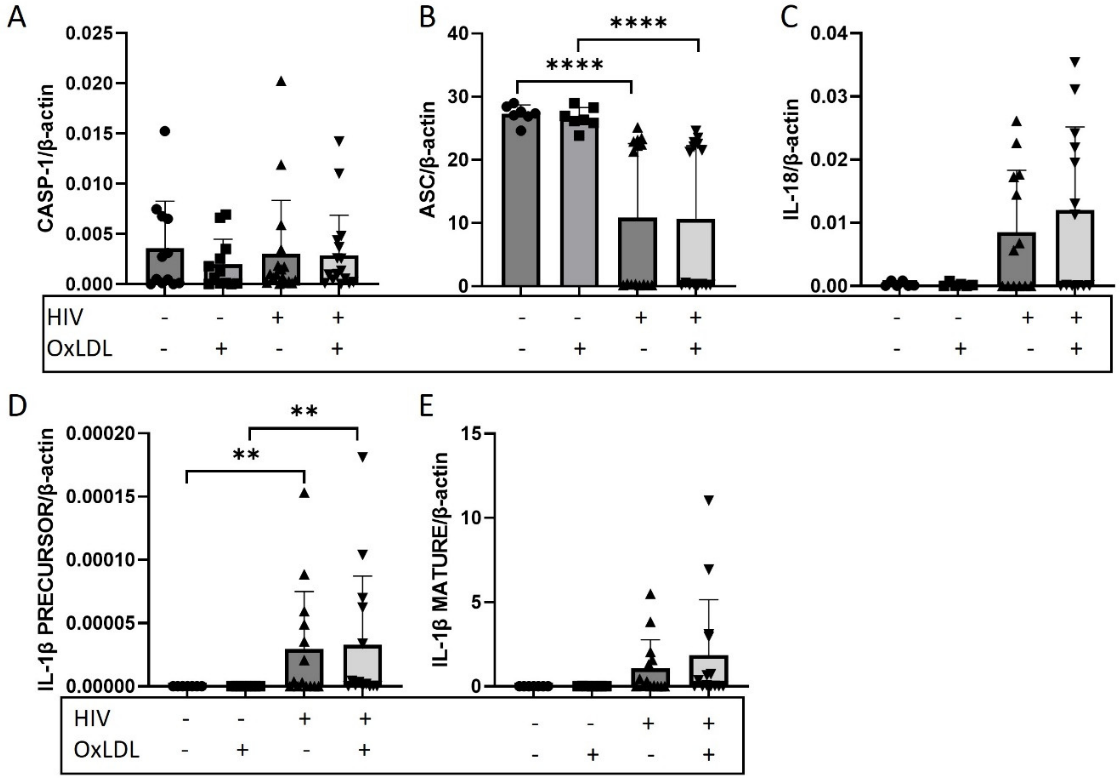

Supplemental Figure S6. qPCRs from PWH and oxLDL treated PBMCs.

Expression of caspase-1 (A), ASC (B), IL-18 (C), IL-1β precursor (D) and mature form (E) measured by qPCR.
